# Genetic characterization of *bla*_NDM_-harboring plasmids in carbapenem-resistant *Escherichia coli* from Myanmar

**DOI:** 10.1371/journal.pone.0184720

**Published:** 2017-09-14

**Authors:** Yo Sugawara, Yukihiro Akeda, Noriko Sakamoto, Dan Takeuchi, Daisuke Motooka, Shota Nakamura, Hideharu Hagiya, Norihisa Yamamoto, Isao Nishi, Hisao Yoshida, Kazuhisa Okada, Khwar Nyo Zin, Mya Mya Aye, Kazunori Tonomo, Shigeyuki Hamada

**Affiliations:** 1 Thailand–Japan Research Collaboration Center on Emerging and Re-emerging Infections, Research Institute for Microbial Diseases, Osaka University, Suita, Japan; 2 Division of Infection Control and Prevention, Osaka University Hospital, Suita, Japan; 3 Department of Infection Metagenomics, Research Institute for Microbial Diseases, Osaka University, Suita, Japan; 4 Laboratory for Clinical Investigation, Osaka University Hospital, Suita, Japan; 5 Clinical Laboratory Department, Yangon General Hospital, Yangon, Myanmar; 6 Bacteriology Research Division, Department of Medical Research, Yangon, Myanmar; Kaohsiung Medical University, TAIWAN

## Abstract

The bacterial enzyme New Delhi metallo-β-lactamase hydrolyzes almost all β-lactam antibiotics, including carbapenems, which are drugs of last resort for severe bacterial infections. The spread of carbapenem-resistant *Enterobacteriaceae* that carry the New Delhi metallo-β-lactamase gene, *bla*_NDM_, poses a serious threat to public health. In this study, we genetically characterized eight carbapenem-resistant *Escherichia coli* isolates from a tertiary care hospital in Yangon, Myanmar. The eight isolates belonged to five multilocus-sequence types and harbored multiple antimicrobial-resistance genes, resulting in resistance against nearly all of the antimicrobial agents tested, except colistin and fosfomycin. Nine plasmids harboring *bla*_NDM_ genes were identified from these isolates. Multiple *bla*_NDM_ genes were found in the distinct Inc-replicon types of the following plasmids: an IncA/C_2_ plasmid harboring *bla*_NDM-1_ (*n* = 1), IncX3 plasmids harboring *bla*_NDM-4_ (*n* = 2) or *bla*_NDM-7_ (*n* = 1), IncFII plasmids harboring *bla*_NDM-4_ (*n* = 1) or *bla*_NDM-5_ (*n* = 3), and a multireplicon F plasmid harboring *bla*_NDM-5_ (*n* = 1). Comparative analysis highlighted the diversity of the *bla*_NDM_-harboring plasmids and their distinct characteristics, which depended on plasmid replicon types. The results indicate circulation of phylogenetically distinct strains of carbapenem-resistant *E*. *coli* with various plasmids harboring *bla*_NDM_ genes in the hospital.

## Introduction

Carbapenems are broad-spectrum antibiotics used as the last line of defense against multidrug-resistant bacteria; however, infections with carbapenem-resistant *Enterobacteriaceae* (CRE) have been increasingly reported since the early 2000s [1], [2]. CRE are resistant to most commonly prescribed antibiotics; therefore, CRE infections are associated with poor prognosis and pose a serious threat in clinical settings [3]. Carbapenem resistance in *Enterobacteriaceae* is primarily due to carbapenemases; plasmid-borne β-lactamases that hydrolyze carbapenems [4]. Several types of carbapenemases, such as KPC, OXA-48, VIM, IMP, and New Delhi metallo-β-lactamase (NDM), have been identified worldwide [5].

Routine worldwide surveillance is essential to understand and prevent CRE transmission; however, in many countries, including Myanmar, surveillance is limited or non-existent. Recently, Myat *et al*. reported the isolation of three *Escherichia coli* and three *Klebsiella pneumoniae* strains harboring *bla*_NDM_ genes in a screening of 592 blood cultures in three hospitals in Yangon, Myanmar [6]. No other carbapenemase-encoding genes were identified in their study, suggesting that NDM rather than other carbapenemases is prevalent in Myanmar. The *bla*_NDM_ genes are usually located adjacent to or in between mobile genetic elements, including transposons and insertion sequences, which facilitate transposition between replicons [7], [8]. Accordingly, *bla*_NDM_ genes are currently found in an array of plasmid replicon types, such as IncF, IncX3, IncL/M, and IncH, as well as in plasmids with a broad-host-range, including IncA/C_2_. Consequently, the *bla*_NDM_ gene has spread from the putative original reservoir, *Acinetobacter*, to enteric bacteria [9]. Additionally, accumulation of nucleotide substitutions in *bla*_NDM_ has produced several variants from the originally identified enzyme NDM-1 [10]. Organisms harboring *bla*_NDM_ genes have been detected not only in hospitals, but also in the environment in some Asian countries [11], [12]. Therefore, it is necessary to characterize the bacterial host, the *bla*_NDM_-harboring plasmid, and the genetic environment associated with *bla*_NDM_ in order to understand the gene acquisition mechanism, track its spread, and investigate possible preventive measures [9].

In this study, we genetically characterized eight carbapenem-resistant *E*. *coli* isolates from a tertiary care hospital in Yangon, Myanmar, using whole-genome sequencing (WGS). Comparative analysis highlighted the diversity of nine plasmids carrying *bla*_NDM_, providing insight into an evolutionary relationship with the already identified *bla*_NDM_ plasmids.

## Materials and methods

### Bacterial isolates

*E*. *coli* isolates were obtained from clinical blood or urine specimens collected from patients at Yangon General Hospital, Yangon, Myanmar, from April to August 2015. Ethical approval for the collection of patient specimens was obtained from the Ethics Committee of Osaka University Graduate School of Medicine and the Department of Medical Research, Myanmar, with a waiver of informed consent. All samples were anonymized before analysis. Specimens were cultured on blood agar plates at 37°C overnight, and each single colony formed was subjected to species identification and antimicrobial-susceptibility testing using a VITEK2 automated system (Sysmex bioMérieux, Marcy l’Etoile, France). *Enterobacteriaceae* strains with minimum inhibitory concentration (MIC) > 2 μg/mL for meropenem were collected and stored at −80°C in phosphate-buffered saline supplemented with 25% glycerol. Isolates were also grown on M-ECC [13], CHROMagar ECC (CHROMagar Microbiology, Paris, France) supplemented with 0.25 μg/mL meropenem and 70 μg/mL ZnSO_4_, and analyzed using an API 20E system (Sysmex bioMérieux) and an EIKEN dry plate (Eiken Chemical, Tokyo, Japan) to reconfirm species identity and antimicrobial susceptibility.

### WGS analysis

Isolates were subjected to WGS using PacBio RSII (Pacific Biosciences, Menlo Park, CA, USA) and MiSeq systems (Illumina, San Diego, CA, USA). To prepare genomic DNA, bacterial isolates were cultured overnight in brain-heart-infusion broth (BD Bacto, Franklin Lakes, NJ, USA) supplemented with 0.25 μg/mL meropenem, and DNA was isolated using a PowerSoil DNA isolation kit (MoBio Laboratories, Carlsbad, CA, USA). To prepare libraries for PacBio RSII, 10 μg DNA was sheared using g-TUBE (Covaris, Woburn, MA, USA) to obtain 10,000–20,000-bp fragments, which were ligated to SMRTbell adapters (Pacific Biosciences). The fragments were processed with a DNA template prep kit 1.0 and a DNA/polymerase binding kit (P6v2; Pacific Biosciences) according to manufacturer’s instructions. Then, the fragments were sequenced with a DNA sequencing kit C4 (Pacific Biosciences), and the sequence reads were assembled *de novo* using HGAP version 2.1.0 with default parameters [14]. The sequences were submitted to the DDBJ/Genbank/EMBL database under accession numbers DRX064631 (M105), DRX064632 (M107), DRX064633 (M109), DRX064634 (M110), DRX064635 (M213), DRX064636 (M214), DRX064638 (M216), and DRX064639 (M217). To prepare libraries for MiSeq analysis, 200 ng of genomic DNA was sheared using Covaris S220 (Covaris) and processed with KAPA library preparation kits (KAPA Biosystems, Boston, MA, USA). Paired-end sequencing with 250-bp reads was performed using a MiSeq version 2 500 cycle kit (Illumina). Reads were mapped to the sequence generated from PacBio RSII using mapping software in CLC Genomics Workbench version 7.5 (CLC Bio, Aarhus, Denmark), and the consensus sequence was extracted for further analysis. The plasmid sequences were submitted to the DDBJ/Genbank/EMBL database under accession numbers AP018136 (pM105_FII), AP018137 (pM105_mF), AP018138 (pM107_FII), AP018139 (pM109_FII), AP018140 (pM110_FII), AP018141 (pM110_X3), AP018142 (pM213_X3), AP018143 (pM214_AC_2_), AP018144 (pM214_FII), AP018145 (pM216_AC_2_), AP018146 (pM216_X3), and AP018147 (pM217_FII).

A phylogenetic tree of the *E*. *coli* isolates based on single-nucleotide polymorphisms (SNPs) was constructed using CSI Phylogeny 1.3 [15]. Multilocus-sequence typing, plasmid-replicon typing, and identification of resistance genes were carried out using MLST 1.8 [16], PlasmidFinder 1.3 [17], and ResFinder 2.1 [18], respectively. Genomic sequences were annotated with RASTtk [19] and MiGAP [20], and genetic structure was compared in EasyFig [21]. A maximum-likelihood tree for IncFII plasmids was constructed using MEGA7 [22].

### Bacterial conjugation and transformation

All *bla*_NDM_-harboring plasmids identified in this study were transferred to laboratory strains by conjugation or transformation and the presence of *bla*_NDM_ and the plasmid-replicon type was confirmed by PCR [23–25].

Bacterial conjugation was carried out as described previously [26], with some modifications. Luria-Bertani (LB) broth cultures of *E*. *coli* isolates in the early exponential phase were mixed with the recipient strain, *E*. *coli* ML4909 [27], at a 1:10 ratio. The bacterial mixture was pelleted by centrifugation, transferred onto nitrocellulose membranes on an LB agar plate, and incubated at 37°C for 6 h. Transconjugants were selected on the LB plate supplemented with 2 μg/mL meropenem and 100 μg/mL rifampicin.

For transformation, plasmids were extracted from overnight cultures of the isolates using the Plasmid Midi kit (Qiagen, Hilden, Germany). HST08 (Takara Bio, Shiga, Japan), a derivative of *E*. *coli* K12, was electroporated with the extracted plasmids using a Gene Pulser Xcell System (Bio-Rad, Hercules, CA, USA), and transformants were selected on brain-heart-infusion agar (BD Bacto) supplemented with 0.25 μg/mL meropenem.

## Results and discussion

### Characterization of carbapenem-resistant *E*. *coli*

**Fig 1:**
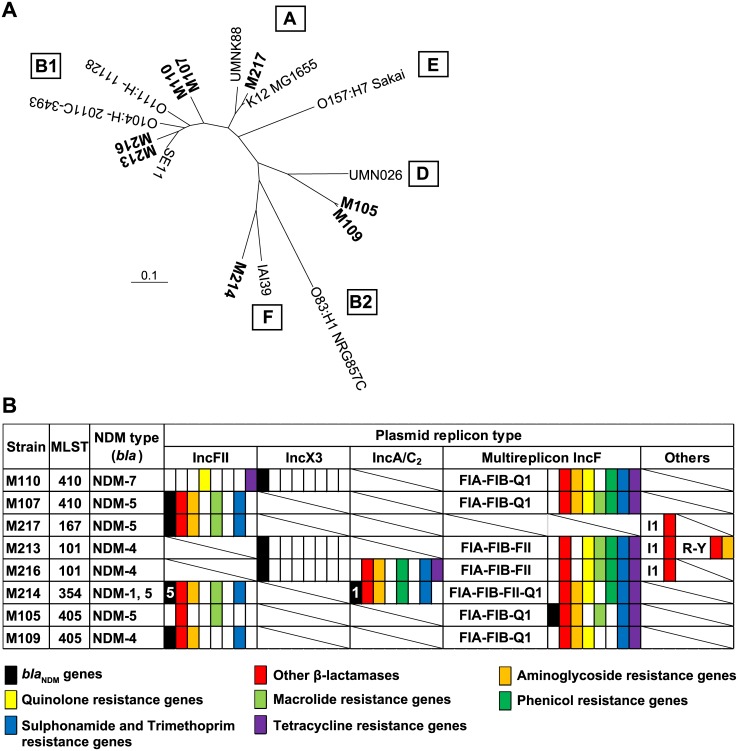
Phylogeny of carbapenem-resistant *E*. *coli* isolated in Myanmar and features of their *bla*_NDM_-harboring plasmids. (A) Phylogenetic tree of carbapenem-resistant *E*. *coli* isolates based on WGS. Reference genomes and several other available genomes were included for comparison. Phylogroups of the reference strains are indicated in boxes. (B) Multilocus-sequence types, NDM types, plasmid-replicon types, and antimicrobial-resistance profiles determined by database search. In strain M214, NDM types are indicated by numbers on black-shaded boxes.

Carbapenem-resistant *E*. *coli* strains (*n* = 8) were isolated at Yangon General Hospital, Yangon, Myanmar, from April to August 2015. Patients that tested positive for carbapenem-resistant *E*. *coli* were admitted to the following wards: hematology (*n* = 5), surgery (*n* = 2), and physical medicine (*n* = 1). Six isolates were obtained from blood specimens derived from patients in the hematology (M105, M109, M110, M214, and M217) and physical medicine (M107) wards, and two were isolated from urine derived from patients in the surgery ward (M213 and M216). All eight isolates were non-susceptible to not only β-lactams, including carbapenems, but also most other antibiotics tested, including aminoglycoside, quinolone, and chloramphenicol (Table 1). Interestingly, isolates from urine were highly resistant to chloramphenicol (MIC > 128), unlike isolates from blood (MIC: 8–32). Colistin appeared to be the only drug effective against all of the isolates examined, with fosfomycin representing a viable alternative for all strains, except for M110 (MIC: 16). By database searches using ResFinder and PlasmidFinder, all isolates were found to carry different types of plasmids encoding several antimicrobial-resistance determinants, including β-lactamases, supporting their multidrug-resistance phenotype (Fig 1B and S1 Table). Phylogenetic analysis based on genome-wide SNPs showed that the isolates belong to different previously defined phylogroups [28], with some belonging to pathogenic phylogroups D and F and others belonging to the commensal and less virulent phylogroups A and B1 (Fig 1A). The isolates were further classified into five multilocus-sequence types; no predominant sequence types were found (Fig 1B). Notably, the five isolates from the hematology ward are not closely related genetically, except for M105 and M109, indicating that different resistant strains disseminated in the ward. M105 and M109 differ in the types of NDM and *bla*_NDM_-harboring plasmid (Fig 1B), suggesting that they had acquired the plasmids independently. This is in contrast to clonal expansion of other drug-resistant bacteria, such as methicillin-resistant *Staphylococcus aureus*, that cause nosocomial infections. A database search with WGS and Sanger-sequencing sequence data indicated that the isolates harbor different *bla*_NDM_ variants (*bla*_NDM-1_, *bla*_NDM-4_, *bla*_NDM-5_, and *bla*_NDM-7_) (Fig 1B) carried by nine distinct plasmids, of which eight belong to the known replicon types IncA/C_2_ (*n* = 1), IncX3 (*n* = 3), and IncFII (*n* = 4). The remaining plasmid is comprised of replication genes of three replicon types (IncFIA, FIB, and Q1). Additionally, isolate M214 harbors two different types of *bla*_NDM_-carrying plasmids (IncFII and A/C_2_).

**Table 1:** MICs of antimicrobial agents for *E*. *coli* clinical isolates from Myanmar.

	MICs (μg/ml) for strain:							
**Antimicrobial**	M105	M107	M109	M110	M213	M214	M216	M217
**Meropenem**	8	4	8	4	>16	>16	8	8
**Doripenem**	8	2	4	4	>16	>16	>16	8
**Imipenem**	4	2	4	4	8	>16	>16	8
**Ceftazidime**	>64	>64	>64	>64	>64	>64	>64	>64
**Cefmetazole**	>32	>32	>32	>32	>32	>32	>32	>32
**Ampicillin/SBT**^a^	>64/128	>64/128	>64/128	>64/128	>64/128	>64/128	>64/128	>64/128
**Piperacillin/TAZ**^a^	>4/512	>4/512	>4/512	>4/512	>4/512	>4/512	>4/512	>4/512
**Aztreonam**	8	>32	>32	>32	>32	32	>32	32
**Gentamicin**	>8	>8	>8	>8	>8	>8	>8	>8
**Amikacin**	>32	>32	>32	≦4	>32	>32	>32	>32
**Ciprofloxacin**	>2	>2	>2	>2	>2	>2	>2	>2
**Levofloxacin**	>4	>4	>4	>4	>4	>4	>4	>4
**Chloramphenicol**	32	8	16	16	>128	16	>128	16
**Minocycline**	>8	>8	>8	>8	>8	>8	>8	2
**ST**^a^	>2/38	>2/38	>2/38	≦2/38	>2/38	>2/38	>2/38	>2/38
**Colistin**	≦0.25	≦0.25	≦0.25	0.5	0.5	1	0.5	0.5
**Fosfomycin**	≦4	≦4	≦4	16	≦4	≦4	≦4	≦4

^a^ SBT; Sulbactam, TAZ; Tazobactam, ST; Sulfamethoxazole/Trimethoprim

### IncA/C_2_ plasmids

**Fig 2:**
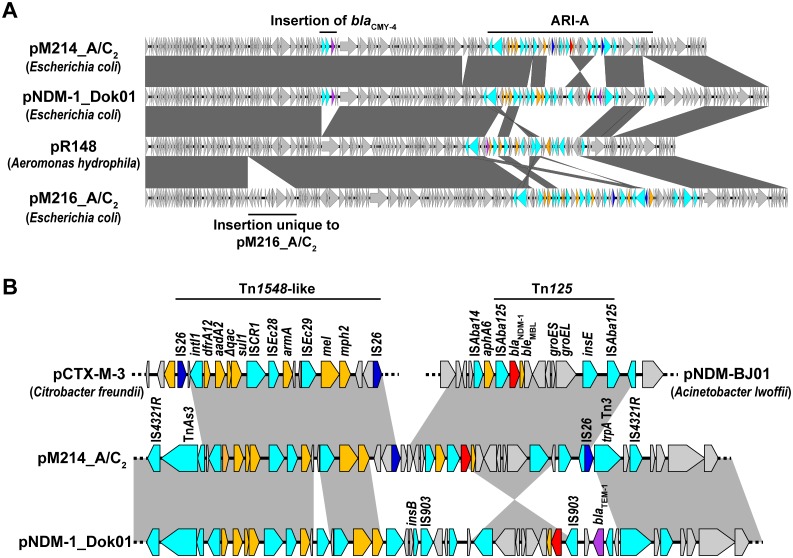
IncA/C_2_ plasmids harboring *bla*_NDM-1_. (A) Comparison of IncA/C_2_ plasmids isolated in this study with similar and/or putative backbone plasmids pNDM-1_Dok01 and pR148. Homologous regions are shaded dark gray. Genes are indicated in colors as follows: *bla*_NDM-1_, red; other β-lactamases, purple; other antimicrobial-resistance genes, orange; IS*26*, blue; other mobile genetic elements, cyan; other accessory genes, light gray. The origins of the indicated plasmids are shown in parentheses. pM214_ A/C_2_ and pNDM-1_Dok01 harbor the class C β-lactamase gene, *bla*_CMY-4_. (B) Schematic representation of the genetic context of *bla*_NDM-1_ in pM214_ A/C_2_ and in other plasmids.

IncA/C_2_ plasmids are broad-host-range plasmids that are predominantly found among NDM-1 producers [29,30]. Indeed, a plasmid we designated as pM214_A/C_2_ represents an IncA/C_2_ plasmid harboring *bla*_NDM-1_ (Fig 2A). A BLAST search indicated that pM214_A/C_2_ shares the highest degree of similarity with pNDM-1_Dok01 (GenBank accession no. AP012208.1), a *bla*_NDM-1_ plasmid isolated from a clinical *E*. *coli* isolate in Japan, but of Indian origin, with 99% identity and 93% query coverage [31]. Both plasmids encode *bla*_NDM-1_ in the ARI-A region, a resistance island designated for IncA/C_2_ [32]; however, the gene clusters containing *bla*_NDM-1_ are inverted between the two plasmids. In pM214_A/C_2_, the resistance island consists of two parts: an IS*Aba125*-mediated composite transposon (Tn*125*) and a Tn*1548*-like transposon. The former region, which harbors *bla*_NDM-1_, completely matches that in pNDM-BJ01 (GenBank accession no. JQ001791.1) (Fig 2B), which was isolated from a clinical isolate of *Acinetobacter lwofii* [33]. Notably, Tn*125* is often detected in *Acinetobacter*; however, the intact form of this transposon has never been found in *Enterobacteriaceae* [34]. This gene cluster is bracketed by IS*26*-insertion sequences in pM214_ A/C_2_, suggesting that the plasmid acquired *bla*_NDM-1_ from pNDM-BJ01 or a closely related plasmid via a single IS*26*-mediated gene transfer. Additionally, the Tn*1548*-like transposon is located adjacent to Tn*125* (Fig 2B). This 15,525-bp fragment contains a class 1 integron comprising several antimicrobial-resistance genes and is identical to an unrelated plasmid (pCTX-M-3, GenBank accession no. AF550415.2) [35], except for one nucleotide. This cluster is partially conserved in other IncA/C_2_ plasmids, such as pNDM-1_Dok01 and pM216_A/C_2_, isolated from strain M216. Therefore, a Tn*1548*-like transposon might have contributed to the ARI-A island in the putative ancestor of these plasmids, with prototypic features being well conserved in pM214_A/C_2_. However, pM216_A/C_2_ encodes neither *bla*_NDM-1_ nor *bla*_CMY-4_, both of which are present in pM214_A/C_2_, but not in the putative precursor plasmid pR148 (GenBank accession no. JX141473.1) [36]. Moreover, the gene array in the ARI-A resistance island significantly differs from that in pM214_A/C_2_ (Fig 2A), suggesting that these two plasmids were derived from different precursors.

### IncX3 plasmids

**Fig 3:**
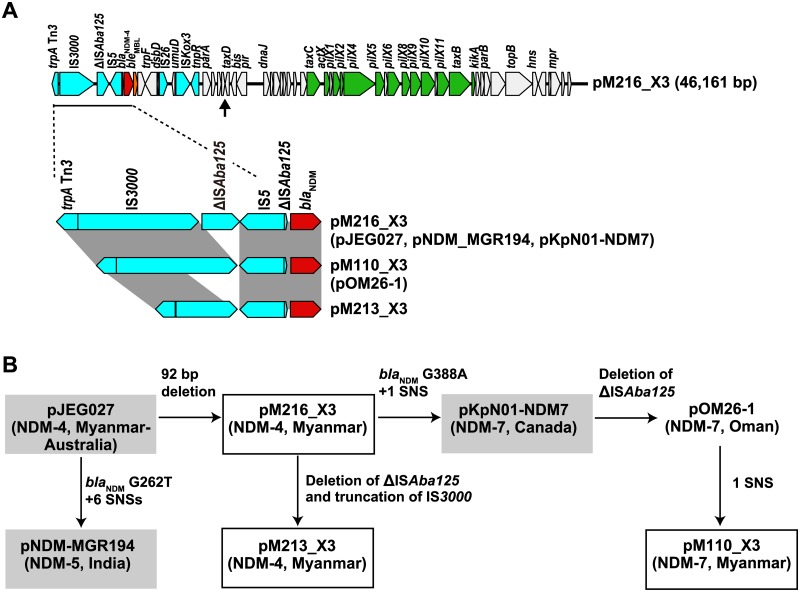
IncX3 plasmids harboring *bla*_NDM_. (A) Schematic representation of the *bla*_NDM-4_ plasmid pM216_X3 and comparison of the genetic context of *bla*_NDM_ in IncX3 plasmids. Genes are indicated in colors as defined in Fig 2, except that genes involved in conjugal transfer are depicted in green. A 92-bp deletion that is found in pM110_X3, pM213_X3, pM216_X3, pKpN01-NDM7, and pOM26-1 is indicated by the arrow. (B) Evolutionary relationship of fully sequenced IncX3 plasmids harboring *bla*_NDM_ as inferred from genetic variances. Encoded NDM variants are shown in parentheses along with geographical origin. The plasmids identified in this study are boxed. pJEG027, pNDM-MGR194, and pKpN01-NDM7 were isolated from *K*. *pneumoniae* isolates and are shaded gray. pOM26-1 was isolated from an *E*. *coli* isolate. SNS, single nucleotide substitution.

We detected three IncX3 plasmids encoding NDM variants: the *bla*_NDM-4_-harboring plasmids pM213_X3 and pM216_X3 and the *bla*_NDM-7_-harboring plasmid pM110_X3. The three plasmids are highly similar to previously reported IncX3 plasmids: the *bla*_NDM-4_-harboring plasmid pJEG027 (GenBank accession no. KM400601.1) [37], the *bla*_NDM-5_-harboring plasmid pNDM_MGR194 (KF220657.1) [38], and the *bla*_NDM-7_-harboring plasmids pKpN01-NDM7 (CP012990.1) [39] and pOM26-1 (KP776609.1), suggesting they share a common ancestor (Fig 3A and 3B). Notably, pJEG027 was isolated from a *K*. *pneumoniae* isolate from an Australian patient hospitalized in Myanmar before returning to Australia [37]. In these IncX3 plasmids, the *bla*_NDM_ genes are encoded in three distinct structures with various lengths of upstream insertion sequences as shown in Fig 3A. Additionally, all of the plasmids found in the survey lack a 92-bp fragment upstream of *taxD* as compared with pJEG027 and pNDM_MGR194 (Fig 3A, arrow) and as previously observed in the *bla*_NDM-7_ plasmids pKpN01-NDM7 and pOM26-1 [37]. The IncX3 plasmids do not carry antimicrobial-resistance genes other than *bla*_NDM_ and apparently represent early steps in the evolution and spread of the *bla*_NDM_ gene [38]. Nonetheless, these plasmids encode distinct NDM variants. Therefore, it is likely that *bla*_NDM_ variants have evolved via nucleotide substitutions within the IncX3 plasmid [37], [39]. This process might be driven by selection for stronger resistance, given that NDM-4, -5, and -7 exhibit higher β-lactamase activity than does NDM-1 [40–42].

### IncFII plasmids

**Fig 4:**
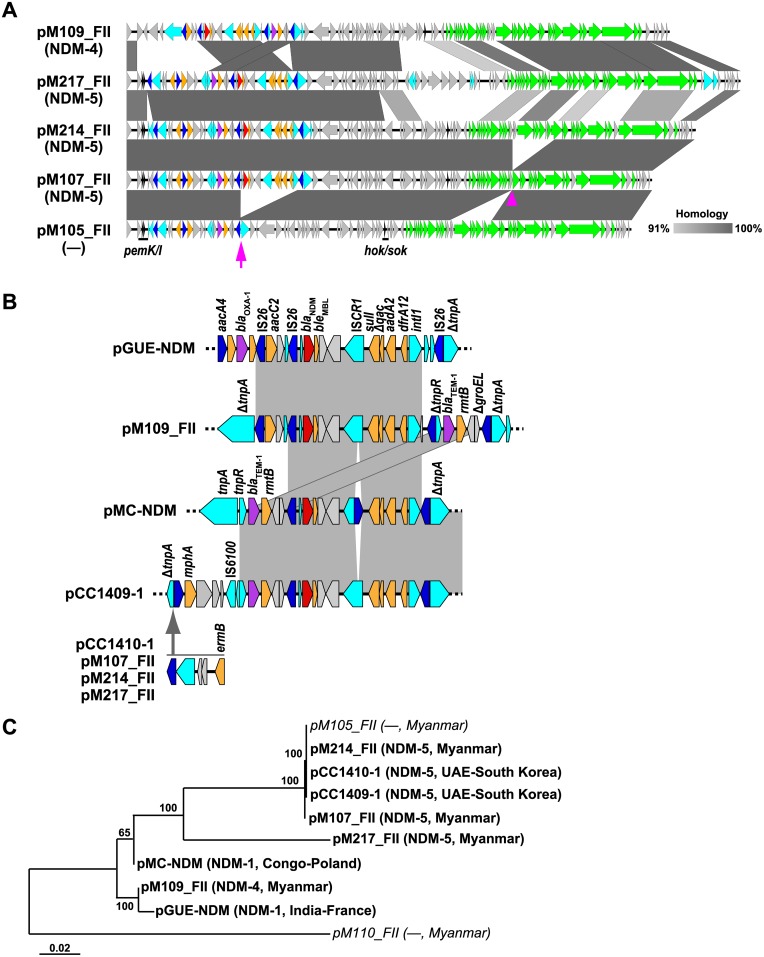
IncFII plasmids harboring *bla*_NDM_. (A) Comparison of IncFII plasmids harboring *bla*_NDM_ in Myanmar isolates. Homologous regions are shaded gray, with the percent identity shaded according to the color bar. The arrowhead indicates a deletion in pM107_FII that encompasses several genes involved in conjugal transfer. The arrow indicates a deletion of a gene cluster carrying *bla*_NDM-5_ in pM105_FII. Genes are indicated in colors as defined in Fig 2, except that genes involved in conjugal transfer and toxin-antitoxin modules are illustrated in green and black, respectively. (B) Schematic representation of the genetic context of *bla*_NDM_. Identical regions are shaded gray, whereas arrows denote gene-cluster insertions. (C) Maximum-likelihood tree of IncFII plasmids harboring *bla*_NDM_. Encoded NDM variants are shown in parentheses along with geographical origin. IncFII plasmids lacking *bla*_NDM_ and found in Myanmar isolates were also included and are indicated in italics. Bootstrap support values based on 1000 replications are indicated at the branching points. All the plasmids, except for pCC1409-1 and pCC1410-1, which were identified in *K*. *pneumoniae* isolates, were isolated from *E*. *coli*.

IncFII was the most predominant type of *bla*_NDM_-harboring plasmid in the isolates investigated. Here, we isolated the *bla*_NDM-4_-harboring plasmid pM109_FII and the *bla*_NDM-5_-harboring plasmids pM107_FII, pM214_FII, and pM217_FII (Fig 4A). In contrast to IncX3 plasmids, these plasmids carry other β-lactamases, as well as resistance genes against several antibiotics, including aminoglycosides, macrolides, sulphonamides, and trimethoprims. The overall genetic structure of these plasmids resembles that of known *bla*_NDM_ IncFII plasmids, such as pGUE-NDM (GenBank accession no. JQ364967.1) [43], pMC-NDM (HG003695.1) [44], and pCC1409-1 (KT725789.1)/pCC1410-1 (KT725788.1) [45], which were found in clinical isolates originating from India, Congo, and United Arab Emirates, respectively. Nevertheless, the genetic structure of the IncFII plasmids is relatively diverse as compared to those of IncX3 plasmids, as we found several insertions/deletions and clear traces of genetic mobilization via IS*26*. pM109_FII, p214_FII, and p217_FII contain a complete transfer operon and were successfully transferred to *E*. *coli* ML4909 by conjugation. However, a fragment was missing from pM107_FII (Fig 4A, arrowhead), rendering the transfer operon incompetent.

Concerning the genetic context of *bla*_NDM_, pM109_FII is distinct from other IncFII Myanmar plasmids, but similar to the *bla*_NDM-1_ plasmid pGUE-NDM (Fig 4B). Both plasmids share a 12-kbp resistance-gene region surrounding *bla*_NDM_. In pM109_FII, an additional gene cassette bracketed by two IS*26* sequences containing the class A β-lactamase gene *bla*_TEM-1_ and the aminoglycoside resistance-coding gene *rmtB* is located downstream of this region. Interestingly, this gene cassette was also found in the *bla*_NDM_ region of other plasmids, including pMC-NDM (Fig 4B). pM109_FII differs from other IncFII Myanmar plasmids in that it lacks the macrolide-resistance gene *ermB* and the toxin-antitoxin module *pemI-pemK* (Fig 4A and 4B).

pM107_FII and pM214_FII clustered with pCC1409-1 and pCC1410-1 in the phylogenetic tree, whereas pM217_FII was assigned to a different branch (Fig 4C). The transfer operon in the former four plasmids differs slightly from that in other IncFII plasmids, with pM214_FII and pM217_FII sharing 81% nucleotide identity in this region. Interestingly, the transfer operon in pM214_FII is completely identical to that in known plasmids, such as pC15-1a [46]. Therefore, the transfer region in pM214_FII and related plasmids might be unrelated to other plasmids, possibly as a result of recombination.

**Fig 5:**
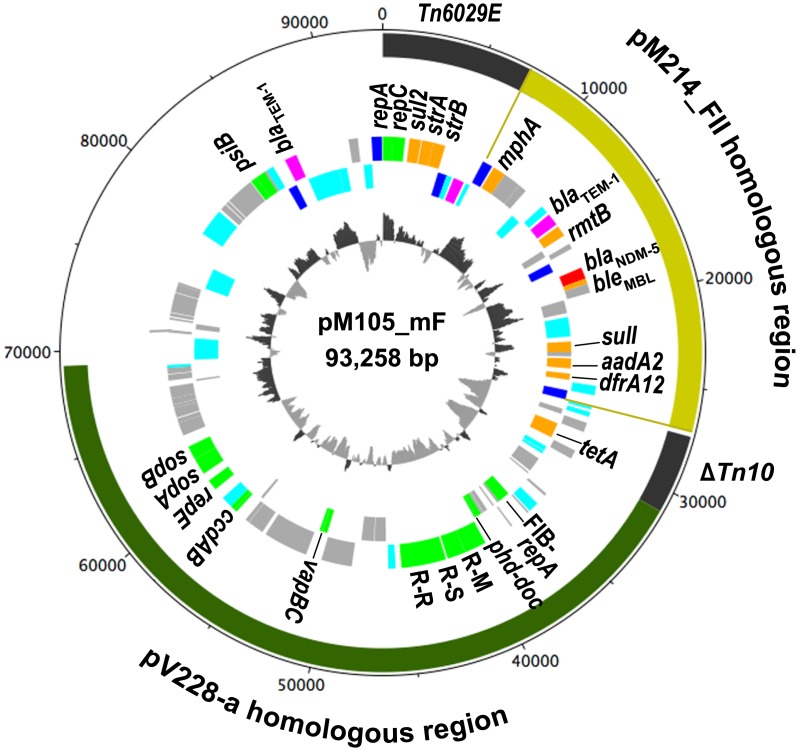
Circular map of plasmid pM105_mF. The outermost circle indicates size in bp. The next circle indicates composite transposons (dark gray) and regions homologous to the *bla*_NDM-5_ region in pM214_FII (yellow) and pV228-a (dark green). The third and fourth circles show coding sequences transcribed clockwise and anti-clockwise, respectively. Genes are indicated in colors as defined in Fig 2, except that genes involved in plasmid replication and maintenance are illustrated in green. R-M, R-S, and R-R denote restriction-modification genes (M, methylase; S, specificity; and R, restriction). The innermost circle shows GC content.

Additionally, we found two IncFII plasmids lacking *bla*_NDM_ (pM110_FII and pM105_FII). pM110_FII encodes resistance genes against quinolone and tetracycline and is phylogenetically distinct from other IncFII plasmids harboring β-lactamases and macrolide-resistance genes (Figs 1B and 4C). pM105_FII, a plasmid identified in strain M105, clustered with the *bla*_NDM-5_-harboring plasmids pM107_FII and pM214_FII. Indeed, pM105_FII is very similar to pM214_FII, except for the region surrounding *bla*_NDM-5_ (Fig 4A, arrow), with 99.3% nucleotide identity outside of this region. Therefore, it is likely that pM105_FII had been encoded previously, but recently lost *bla*_NDM-5_ along with other neighboring genes. This region is bracketed by two IS*26* sequences, implying that IS*26* was involved in the loss of this fragment [47]. Additionally, the M105 isolate encodes *bla*_NDM-5_ on a multireplicon IncF plasmid designated as pM105_mF, and, notably, the genetic context of *bla*_NDM-5_ in this plasmid is identical to that in pM214_FII and related plasmids (Fig 5). Furthermore, the resistance island in pM107_FII and pM214_FII bracketed by two IS*26* sequences is conserved in pM105_mF (Fig 5, yellow). This implies that pM105_mF likely acquired *bla*_NDM-5_ from the pM105_FII precursor or related IncFII plasmids via IS*26*.

### Other plasmids

Multiple replicons might broaden the plasmid host range and enable the maintenance of incompatible replicons [24]. To the best of our knowledge, the pM105_mF plasmid backbone comprising IncFIA-, FIB-, and Q1-replication genes (Fig 1B, designated FIA-FIB-Q1) has not been previously reported. A BLAST search revealed a partial match to pV228-a (Fig 5, dark green), with 99% identity, but query coverage of only 54%. pV228-a, containing IncFIA-, FIB-, and FII-replication genes, has been identified in an *E*. *coli* strain isolated from a sewage-treatment plant in India [48], and similar multireplicon F plasmids have been found in three other Myanmar isolates (Fig 1B), with one containing an IncFII-replication gene (FIA-FIB-FII-Q1). These plasmids commonly encode several antimicrobial-resistance determinants, such as *bla*_TEM-1_, and resistance genes against sulphonamide, trimethoprim, and tetracycline. Collectively, these data indicate that multireplicon plasmids with several antimicrobial-resistance genes might be widespread in Myanmar.

Although it is unclear whether the *bla*_NDM-5_ gene cluster was transferred directly from a precursor of pM105_FII onto pM105_mF, our findings demonstrated that mobilization of *bla*_NDM_ between plasmids resulted in a novel resistance plasmid. Additionally, the number of antimicrobial-resistance genes in pM105_mF was higher than that in IncFII plasmids harboring *bla*_NDM-5_ (Fig 1B and S1 Table), suggesting that recombination increased the number of resistance genes encoded on the same plasmid as *bla*_NDM_. As shown in Fig 1B, several multidrug-resistant plasmids, such as multireplicon F plasmids, co-exist with *bla*_NDM_ plasmids in all isolates. Therefore, clinical settings in Myanmar appear to provide a breeding ground for novel resistance plasmids, and thus require more vigilance from national and local public health authorities.

## Conclusions

We genetically characterized clinical carbapenem-resistant *E*. *coli* isolates from Myanmar and discovered multiple-resistance plasmids and several NDM variants in the diverse genetic backgrounds of the bacteria, even within the eight isolates examined. These findings suggest dissemination of NDM via multiple introductions and/or prolonged presence in the hospital allowing for recombination and dissemination via horizontal gene transfer into various genetic backgrounds. The situation seems to be similar to that reported in India, where NDM is already endemic and is found not only in healthcare settings, but also in the community [11], [49]. Therefore, further surveillance, including both the hospital and the community, is warranted to understand the dissemination of CRE in Myanmar.

## Supporting information

S1 Table Antimicrobial resistance determinants carried by the plasmids identified in *E*. *coli* clinical isolates in Myanmar. (DOCX)

## References

[1] Papp-Wallace KM, Endimiani A, Taracila MA, Bonomo RA. Carbapenems: past, present, and future. Antimicrob Agents Chemother. 2011;55: 4943–4960. doi: 10.1128/AAC.00296-11 21859938 10.1128/AAC.00296-11 PMC3195018

[2] Temkin E, Adler A, Lerner A, Carmeli Y. Carbapenem-resistant Enterobacteriaceae: biology, epidemiology, and management. Ann N Y Acad Sci. 2014;1323: 22–42. doi: 10.1111/nyas.12537 25195939 10.1111/nyas.12537

[3] Falagas ME, Tansarli GS, Karageorgopoulos DE, Vardakas KZ. Deaths attributable to carbapenem-resistant Enterobacteriaceae infections. Emerg Infect Dis. 2014;20:1170–1175. doi: 10.3201/eid2007.121004 24959688 10.3201/eid2007.121004 PMC4073868

[4] Queenan AM, Bush K. Carbapenemases: the versatile beta-lactamases. Clin Microbiol Rev. 2007;20: 440–458. doi: 10.1128/CMR.00001-07 17630334 10.1128/CMR.00001-07 PMC1932750

[5] Logan LK, Weinstein RA. The Epidemiology of Carbapenem-Resistant Enterobacteriaceae: The Impact and Evolution of a Global Menace. J Infect Dis. 2017;215: S28–S36. doi: 10.1093/infdis/jiw282 28375512 10.1093/infdis/jiw282 PMC5853342

[6] Myat TO, Hannaway RF, Zin KN, Htike WW, Crump JA, Murdoch DR, et al ESBL- and Carbapenemase-Producing Enterobacteriaceae in Patients with Bacteremia, Yangon, Myanmar, 2014. Emerg Infect Dis. 2017;23: 857–859. doi: 10.3201/eid2305.161100 28418298 10.3201/eid2305.161100 PMC5403063

[7] Poirel L, Bonnin RA, Boulanger A, Schrenzel J, Kaase M, Nordmann P. Tn*125*-related acquisition of *bla*_NDM_-like genes in *Acinetobacter baumannii*. Antimicrob Agents Chemother. 2012;56: 1087–1089. doi: 10.1128/AAC.05620-11 22143526 10.1128/AAC.05620-11 PMC3264265

[8] Partridge SR, Iredell JR. Genetic contexts of *bla*_NDM-1_. Antimicrob Agents Chemother. 2012;56: 6065–6067. doi: 10.1128/AAC.00117-12 23074228 10.1128/AAC.00117-12 PMC3486571

[9] Wailan AM, Paterson DL. The spread and acquisition of NDM-1: a multifactorial problem. Expert Rev Anti Infect The. 2014;12: 91–115. 10.1586/14787210.2014.856756 24308710

[10] Dortet L, Poirel L, Nordmann P. Worldwide dissemination of the NDM-type carbapenemases in Gram-negative bacteria. Biomed Res Int. 2014;2014: 249856 doi: 10.1155/2014/249856 24790993 10.1155/2014/249856 PMC3984790

[11] Walsh TR, Weeks J, Livermore DM, Toleman MA. Dissemination of NDM-1 positive bacteria in the New Delhi environment and its implications for human health: an environmental point prevalence study. Lancet Infect Dis. 2011;11: 355–362. doi: 10.1016/S1473-3099(11)70059-7 21478057 10.1016/S1473-3099(11)70059-7

[12] Hsu LY, Apisarnthanarak A, Khan E, Suwantarat N, Ghafur A, Tambyah PA. Carbapenem-resistant *Acinetobacter baumannii* and *Enterobacteriaceae* in South and Southeast Asia. Clin Microbiol Rev. 2017;30: 1–22. doi: 10.1128/CMR.00042-16 27795305 10.1128/CMR.00042-16 PMC5217790

[13] Yamamoto N, Kawahara R, Akeda Y, Shanmugakani RK, Yoshida H, Hagiya H, et al Development of selective medium for IMP-type carbapenemase-producing *Enterobacteriaceae* in stool specimens. BMC Infect Dis. 2017;17: 229 doi: 10.1186/s12879-017-2312-1 28340557 10.1186/s12879-017-2312-1 PMC5366124

[14] Chin CS, Alexander DH, Marks P, Klammer AA, Drake J, Heiner C, et al Nonhybrid, finished microbial genome assemblies from long-read SMRT sequencing data. Nat Methods. 2013;10: 563–569. doi: 10.1038/nmeth.2474 23644548 10.1038/nmeth.2474

[15] Kaas RS, Leekitcharoenphon P, Aarestrup FM, Lund O. Solving the problem of comparing whole bacterial genomes across different sequencing platforms. PLoS One. 2014;9: e104984 doi: 10.1371/journal.pone.0104984 25110940 10.1371/journal.pone.0104984 PMC4128722

[16] Larsen MV, Cosentino S, Rasmussen S, Friis C, Hasman H, Marvig RL, et al Multilocus sequence typing of total-genome-sequenced bacteria. J Clin Microbiol. 2012;50:1355–1361. doi: 10.1128/JCM.06094-11 22238442 10.1128/JCM.06094-11 PMC3318499

[17] Carattoli A, Zankari E, García-Fernández A, Voldby Larsen M, Lund O, Villa L, et al *In silico* detection and typing of plasmids using PlasmidFinder and plasmid multilocus sequence typing. Antimicrob Agents Chemother. 2014;58: 3895–3903. doi: 10.1128/AAC.02412-14 24777092 10.1128/AAC.02412-14 PMC4068535

[18] Zankari E, Hasman H, Cosentino S, Vestergaard M, Rasmussen S, Lund O, et al Identification of acquired antimicrobial resistance genes. J Antimicrob Chemother. 2012;67: 2640–2644. doi: 10.1093/jac/dks261 22782487 10.1093/jac/dks261 PMC3468078

[19] Brettin T, Davis JJ, Disz T, Edwards RA, Gerdes S, Olsen GJ, et al RASTtk: a modular and extensible implementation of the RAST algorithm for building custom annotation pipelines and annotating batches of genomes. Sci Rep. 2015;5: 8365 doi: 10.1038/srep08365 25666585 10.1038/srep08365 PMC4322359

[20] Sugawara H, Ohyama A, Mori H, Kurokawa K. Microbial Genome Annotation Pipeline (MiGAP) for diverse users. In: Abtracts of the 20th International Conference on Genome Informatics, Yokohama, 2009. Abstract p. S001-1-2. Yokohama, Japan: Japanese Society for Bioinformatics, Tokyo, Japan.

[21] Sullivan MJ, Petty NK, Beatson SA. Easyfig: a genome comparison visualizer. Bioinformatics. 2011;27: 1009–1010. doi: 10.1093/bioinformatics/btr039 21278367 10.1093/bioinformatics/btr039 PMC3065679

[22] Kumar S, Stecher G, Tamura K. MEGA7: molecular evolutionary genetics analysis version 7.0 for bigger datasets. Mol Biol Evol. 2016;33: 1870–1874. doi: 10.1093/molbev/msw054 27004904 10.1093/molbev/msw054 PMC8210823

[23] Carattoli A, Bertini A, Villa L, Falbo V, Hopkins KL, Threlfall EJ. Identification of plasmids by PCR-based replicon typing. J Microbiol Methods. 2005;63: 219–228. doi: 10.1016/j.mimet.2005.03.018 15935499 10.1016/j.mimet.2005.03.018

[24] Villa L, García-Fernández A, Fortini D, Carattoli A. Replicon sequence typing of IncF plasmids carrying virulence and resistance determinants. J Antimicrob Chemother. 2010;65: 2518–2529. doi: 10.1093/jac/dkq347 20935300 10.1093/jac/dkq347

[25] Johnson TJ, Bielak EM, Fortini D, Hansen LH, Hasman H, Debroy C, et al Expansion of the IncX plasmid family for improved identification and typing of novel plasmids in drug-resistant *Enterobacteriaceae*. Plasmid. 2012;68: 43–50. doi: 10.1016/j.plasmid.2012.03.001 22470007 10.1016/j.plasmid.2012.03.001

[26] Inoue M, Itoh J, Mitsuhashi S. pMS76, a plasmid capable of amplification by treatment with chloramphenicol. Plasmid. 1983;9: 86–97. 6300947 10.1016/0147-619x(83)90033-1

[27] Ma L, Ishii Y, Ishiguro M, Matsuzawa H, Yamaguchi K. Cloning and sequencing of the gene encoding Toho-2, a class A beta-lactamase preferentially inhibited by tazobactam. Antimicrob Agents Chemother. 1998;42: 1181–1186. 9593147 10.1128/aac.42.5.1181 PMC105770

[28] Clermont O, Christenson JK, Denamur E, Gordon DM. The Clermont *Escherichia coli* phylo-typing method revisited: improvement of specificity and detection of new phylo-groups. Environ Microbiol Rep. 2013;5: 58–65. doi: 10.1111/1758-2229.12019 23757131 10.1111/1758-2229.12019

[29] Poirel L, Dortet L, Bernabeu S, Nordmann P. Genetic features of *bla*_NDM-1_-positive *Enterobacteriaceae*. Antimicrob Agents Chemother. 2011;55:5403–5407. doi: 10.1128/AAC.00585-11 21859933 10.1128/AAC.00585-11 PMC3195013

[30] Sartor AL, Raza MW, Abbasi SA, Day KM, Perry JD, Paterson DL, et al Molecular epidemiology of NDM-1-producing *Enterobacteriaceae* and *Acinetobacter baumannii* isolates from Pakistan. Antimicrob Agents Chemother. 2014;58: 5589–5593. doi: 10.1128/AAC.02425-14 24982081 10.1128/AAC.02425-14 PMC4135889

[31] Sekizuka T, Matsui M, Yamane K, Takeuchi F, Ohnishi M, Hishinuma A, et al Complete sequencing of the *bla*_NDM-1_-positive IncA/C plasmid from *Escherichia coli* ST38 isolate suggests a possible origin from plant pathogens. PLoS One. 2011;6: e25334 doi: 10.1371/journal.pone.0025334 21966500 10.1371/journal.pone.0025334 PMC3179503

[32] Harmer CJ, Hall RM. pRMH760, a precursor of A/C₂ plasmids carrying *bla*_CMY_ and *bla*_NDM_ genes. Microb Drug Resist. 2014;20:416–423. doi: 10.1089/mdr.2014.0012 24841670 10.1089/mdr.2014.0012

[33] Hu H, Hu Y, Pan Y, Liang H, Wang H, Wang X, et al Novel plasmid and its variant harboring both a *bla*_NDM-1_ gene and type IV secretion system in clinical isolates of *Acinetobacter lwoffii*. Antimicrob Agents Chemother. 2012;56: 1698–1702. doi: 10.1128/AAC.06199-11 22290961 10.1128/AAC.06199-11 PMC3318331

[34] Chen Z, Li H, Feng J, Li Y, Chen X, Guo X, et al NDM-1 encoded by a pNDM-BJ01-like plasmid p3SP-NDM in clinical *Enterobacter aerogenes*. Front Microbiol. 2015;6: 294 doi: 10.3389/fmicb.2015.00294 25926823 10.3389/fmicb.2015.00294 PMC4396501

[35] Gołebiewski M, Kern-Zdanowicz I, Zienkiewicz M, Adamczyk M, Zylinska J, Baraniak A, et al Complete nucleotide sequence of the pCTX-M3 plasmid and its involvement in spread of the extended-spectrum beta-lactamase gene *bla*_CTX-M-3_. Antimicrob Agents Chemother. 2007;51: 3789–3795. doi: 10.1128/AAC.00457-07 17698626 10.1128/AAC.00457-07 PMC2151408

[36] Del Castillo CS, Hikima J, Jang HB, Nho SW, Jung TS, Wongtavatchai J, et al Comparative sequence analysis of a multidrug-resistant plasmid from *Aeromonas hydrophila*. Antimicrob Agents Chemother. 2013;57:120–129. doi: 10.1128/AAC.01239-12 23070174 10.1128/AAC.01239-12 PMC3535917

[37] Espedido BA, Dimitrijovski B, van Hal SJ, Jensen SO. The use of whole-genome sequencing for molecular epidemiology and antimicrobial surveillance: identifying the role of IncX3 plasmids and the spread of *bla*_NDM-4_-like genes in the Enterobacteriaceae J Clin Pathol. 2015;68:835–838. doi: 10.1136/jclinpath-2015-203044 26056157 10.1136/jclinpath-2015-203044

[38] Krishnaraju M, Kamatchi C, Jha AK, Devasena N, Vennila R, Sumathi G, et al Complete sequencing of an IncX3 plasmid carrying *bla*_NDM-5_ allele reveals an early stage in the dissemination of the *bla*_NDM_ gene. Indian J Med Microbiol. 2015;33: 30–38. doi: 10.4103/0255-0857.148373 25559999 10.4103/0255-0857.148373

[39] Chen L, Peirano G, Lynch T, Chavda KD, Gregson DB, Church DL, et al Molecular characterization by using next-generation sequencing of plasmids containing *bla*_NDM-7_ in *Enterobacteriaceae* from Calgary, Canada. Antimicrob Agents Chemother. 2015;60: 1258–1263. doi: 10.1128/AAC.02661-15 26643346 10.1128/AAC.02661-15 PMC4775971

[40] Nordmann P, Boulanger AE, Poirel L. NDM-4 metallo-β-lactamase with increased carbapenemase activity from *Escherichia coli*. Antimicrob Agents Chemother. 2012;56: 2184–2186. doi: 10.1128/AAC.05961-11 22252797 10.1128/AAC.05961-11 PMC3318389

[41] Cuzon G, Bonnin RA, Nordmann P. First identification of novel NDM carbapenemase, NDM-7, in *Escherichia coli* in France. PLoS One. 2013;8: e61322 doi: 10.1371/journal.pone.0061322 23593461 10.1371/journal.pone.0061322 PMC3625146

[42] Göttig S, Hamprecht AG, Christ S, Kempf VA, Wichelhaus TA. Detection of NDM-7 in Germany, a new variant of the New Delhi metallo-β-lactamase with increased carbapenemase activity. J Antimicrob Chemother. 2013;68: 1737–1740. doi: 10.1093/jac/dkt088 23557929 10.1093/jac/dkt088

[43] Bonnin RA, Poirel L, Carattoli A, Nordmann P. Characterization of an IncFII plasmid encoding NDM-1 from *Escherichia coli* ST131. PLoS One. 2012;7: e34752 doi: 10.1371/journal.pone.0034752 22511964 10.1371/journal.pone.0034752 PMC3325265

[44] Fiett J, Baraniak A, Izdebski R, Sitkiewicz I, Żabicka D, Meler A, et al The first NDM metallo-β-lactamase-producing *Enterobacteriaceae* isolate in Poland: evolution of IncFII-type plasmids carrying the *bla*_NDM-1_ gene. Antimicrob Agents Chemother. 2014;58: 1203–1207. doi: 10.1128/AAC.01197-13 24247128 10.1128/AAC.01197-13 PMC3910837

[45] Shin J, Baek JY, Cho SY, Huh HJ, Lee NY, Song JH, et al *bla*_NDM-5_-bearing IncFII-type plasmids of *Klebsiella pneumoniae* sequence type 147 transmitted by cross-border transfer of a patient. Antimicrob Agents Chemother. 2016;60: 1932–1934. doi: 10.1128/AAC.02722-15 26824953 10.1128/AAC.02722-15 PMC4775946

[46] Boyd DA, Tyler S, Christianson S, McGeer A, Muller MP, Willey BM, et al Complete nucleotide sequence of a 92-kilobase plasmid harboring the CTX-M-15 extended-spectrum beta-lactamase involved in an outbreak in long-term-care facilities in Toronto, Canada. Antimicrob Agents Chemother. 2004;48:3758–3764. doi: 10.1128/AAC.48.10.3758-3764.2004 15388431 10.1128/AAC.48.10.3758-3764.2004 PMC521865

[47] He S, Hickman AB, Varani AM, Siguier P, Chandler M, Dekker JP, et al Insertion sequence IS26 reorganizes plasmids in clinically isolated multidrug-resistant bacteria by replicative transposition. MBio. 2015;6: e00762 doi: 10.1128/mBio.00762-15 26060276 10.1128/mBio.00762-15 PMC4471558

[48] Akiba M, Sekizuka T, Yamashita A, Kuroda M, Fujii Y, Murata M, et al Distribution and relationships of antimicrobial resistance determinants among extended-spectrum-cephalosporin-resistant or carbapenem-resistant *Escherichia coli* isolates from rivers and sewage treatment plants in India. Antimicrob Agents Chemother. 2016;60: 2972–2980. doi: 10.1128/AAC.01950-15 26953207 10.1128/AAC.01950-15 PMC4862527

[49] Rahman M, Shukla SK, Prasad KN, Ovejero CM, Pati BK, Tripathi A, et al Prevalence and molecular characterisation of New Delhi metallo-β-lactamases NDM-1, NDM-5, NDM-6 and NDM-7 in multidrug-resistant Enterobacteriaceae from India. Int J Antimicrob Agents. 2014;44: 30–37. doi: 10.1016/j.ijantimicag.2014.03.003 24831713 10.1016/j.ijantimicag.2014.03.003

