# Correction: Genetic characterization of *bla*_NDM_-harboring plasmids in carbapenem-resistant *Escherichia coli* from Myanmar

**DOI:** 10.1371/journal.pone.0189435

**Published:** 2017-12-06

**Authors:** The PLOS ONE Staff

The fourteenth author's name is spelled incorrectly. The correct name is: Kazunori Tomono. The publisher apologizes for this error.

## References

[1] Sugawara Y, Akeda Y, Sakamoto N, Takeuchi D, Motooka D, Nakamura S, et al (2017) Genetic characterization of *bla*_NDM_-harboring plasmids in carbapenem-resistant *Escherichia coli* from Myanmar. PLoS ONE 12(9): e0184720 https://doi.org/10.1371/journal.pone.0184720 28910381 10.1371/journal.pone.0184720 PMC5598989

