# Supplementary material for: Genetic characterization of blaNDM-harboring plasmids in carbapenem-resistant Escherichia coli from Myanmar
Source: PLoS One. 2017 Sep 14;12(9):e0184720. doi: 10.1371/journal.pone.0184720 (PMC5598989; doi:10.1371/journal.pone.0184720)
Supplement: S1 Table — (DOCX) [file pone.0184720.s001.docx]

**S1 Table.** Antimicrobial resistance determinants carried by the plasmids identified in *E. coli* clinical isolates in Myanmar.

|  | | Antimicrobial resistance determinant determined by ResFinder | | | | | | | | | | | | | | | | | | | | | | | | | | | | | | | | | | |
| --- | --- | --- | --- | --- | --- | --- | --- | --- | --- | --- | --- | --- | --- | --- | --- | --- | --- | --- | --- | --- | --- | --- | --- | --- | --- | --- | --- | --- | --- | --- | --- | --- | --- | --- | --- | --- |
|  |  | β-lactamase (*bla*) | | | | | Aminoglycoside resistance gene | | | | | | | | | | | | | Macrolide resistance gene | | | | Phenicol resistance gene | | Quinolone resistance gene | | | Sulphonamide/ Trimethoprim resistance gene | | | | | Tetracycline resistance gene | | |
|  |  | NDM* | TEM-1B | CTX-M-15 | CMY* | OXA-1 | *armA* | *rmtB* | *aac(3)-IIa* | *aac(3)-IId* | *aac(6’)-IIa* | *aac(6’)Ib-cr* | *aph(3’)-Ia* | *aph(3’)-Ic* | *aph(3’)-VIa* | *strA, strB* | *aadA2* | *aadA4* | *aadA5* | *ermB* | *mphA* | *mphE* | *msrE* | *catA1* | *catB3* | *qnrS1* | *qepA* | *aac(6’)Ib-cr* | *sul1* | *sul2* | *dfrA12* | *dfrA17* | *dfrB4* | *tetA* | *tetB* | *tetD* |
| Strain | Plasmid replicon type determined by PlasmidFinder (Inc) |  |  |  |  |  |  |  |  |  |  |  |  |  |  |  |  |  |  |  |  |  |  |  |  |  |  |  |  |  |  |  |  |  |  |  |
| M105 | FIA-FIB-Q1 | **5** |  |  |  |  |  |  |  |  |  |  |  |  |  |  |  |  |  |  |  |  |  |  |  |  |  |  |  |  |  |  |  |  |  |  |
|  | FII |  |  |  |  |  |  |  |  |  |  |  |  |  |  |  |  |  |  |  |  |  |  |  |  |  |  |  |  |  |  |  |  |  |  |  |
| M107 | FIA-FIB-Q1 |  |  |  |  |  |  |  |  |  |  |  |  |  |  |  |  |  |  |  |  |  |  |  |  |  |  |  |  |  |  |  |  |  |  |  |
|  | FII | **5** |  |  |  |  |  |  |  |  |  |  |  |  |  |  |  |  |  |  |  |  |  |  |  |  |  |  |  |  |  |  |  |  |  |  |
| M109 | FIA-FIB-Q1 |  |  |  |  |  |  |  |  |  |  |  |  |  |  |  |  |  |  |  |  |  |  |  |  |  |  |  |  |  |  |  |  |  |  |  |
|  | FII | **4** |  |  |  |  |  |  |  |  |  |  |  |  |  |  |  |  |  |  |  |  |  |  |  |  |  |  |  |  |  |  |  |  |  |  |
| M110 | FIA-FIB-FII-Q1 |  |  |  |  |  |  |  |  |  |  |  |  |  |  |  |  |  |  |  |  |  |  |  |  |  |  |  |  |  |  |  |  |  |  |  |
|  | FII |  |  |  |  |  |  |  |  |  |  |  |  |  |  |  |  |  |  |  |  |  |  |  |  |  |  |  |  |  |  |  |  |  |  |  |
|  | X3 | **7** |  |  |  |  |  |  |  |  |  |  |  |  |  |  |  |  |  |  |  |  |  |  |  |  |  |  |  |  |  |  |  |  |  |  |
| M213 | FIA-FIB-FII |  |  |  |  |  |  |  |  |  |  |  |  |  |  |  |  |  |  |  |  |  |  |  |  |  |  |  |  |  |  |  |  |  |  |  |
|  | I1 |  |  |  | **42** |  |  |  |  |  |  |  |  |  |  |  |  |  |  |  |  |  |  |  |  |  |  |  |  |  |  |  |  |  |  |  |
|  | R-Y |  |  |  |  |  |  |  |  |  |  |  |  |  |  |  |  |  |  |  |  |  |  |  |  |  |  |  |  |  |  |  |  |  |  |  |
|  | X3 | **4** |  |  |  |  |  |  |  |  |  |  |  |  |  |  |  |  |  |  |  |  |  |  |  |  |  |  |  |  |  |  |  |  |  |  |
| M214 | A/C_2_ | **1** |  |  | **4** |  |  |  |  |  |  |  |  |  |  |  |  |  |  |  |  |  |  |  |  |  |  |  |  |  |  |  |  |  |  |  |
|  | FIA-FIB-FII-Q1 |  |  |  |  |  |  |  |  |  |  |  |  |  |  |  |  |  |  |  |  |  |  |  |  |  |  |  |  |  |  |  |  |  |  |  |
|  | FII | **5** |  |  |  |  |  |  |  |  |  |  |  |  |  |  |  |  |  |  |  |  |  |  |  |  |  |  |  |  |  |  |  |  |  |  |
| M216 | A/C_2_ |  |  |  |  |  |  |  |  |  |  |  |  |  |  |  |  |  |  |  |  |  |  |  |  |  |  |  |  |  |  |  |  |  |  |  |
|  | FIA-FIB-FII |  |  |  |  |  |  |  |  |  |  |  |  |  |  |  |  |  |  |  |  |  |  |  |  |  |  |  |  |  |  |  |  |  |  |  |
|  | I1 |  |  |  | **42** |  |  |  |  |  |  |  |  |  |  |  |  |  |  |  |  |  |  |  |  |  |  |  |  |  |  |  |  |  |  |  |
|  | X3 | **4** |  |  |  |  |  |  |  |  |  |  |  |  |  |  |  |  |  |  |  |  |  |  |  |  |  |  |  |  |  |  |  |  |  |  |
| M217 | FII | **5** |  |  |  |  |  |  |  |  |  |  |  |  |  |  |  |  |  |  |  |  |  |  |  |  |  |  |  |  |  |  |  |  |  |  |
|  | I1 |  |  |  | **42** |  |  |  |  |  |  |  |  |  |  |  |  |  |  |  |  |  |  |  |  |  |  |  |  |  |  |  |  |  |  |  |

*Types of *bla*_NDM_ or *bla*_CMY_ are indicated by numbers on black-shaded boxes.
